# Broad application of a simple and affordable protocol for isolating plant RNA

**DOI:** 10.1186/s13104-015-1119-7

**Published:** 2015-04-16

**Authors:** Daniel Couto, Lena Stransfeld, Ana Arruabarrena, Cyril Zipfel, Rosa Lozano-Durán

**Affiliations:** The Sainsbury Laboratory, Norwich Research Park, Norwich, NR4 7UH United Kingdom; National Agricultural Research Institute (INIA), Estación Experimental INIA Salto Grande, Salto, CP50000 Uruguay; Shanghai Institutes of Biological Sciences, Shanghai Center for Plant Stress Biology (PSC), Chinese Academy of Sciences, Shanghai, 201602 China

**Keywords:** RNA, Arabidopsis, Tomato, Wheat, Affordable, Protocol

## Abstract

**Background:**

Standard molecular biological methods involve the analysis of gene expression in living organisms under diverse environmental and developmental conditions. One of the most direct approaches to quantify gene expression is the isolation of RNA. Most techniques used to quantify gene expression require the isolation of RNA, usually from a large number of samples. While most published protocols, including those for commercial reagents, are either labour intensive, use hazardous chemicals and/or are costly, a previously published protocol for RNA isolation in *Arabidopsis thaliana* yields high amounts of good quality RNA in a simple, safe and inexpensive manner.

**Findings:**

We have tested this protocol in tomato and wheat leaves, as well as in Arabidopsis leaves, and compared the resulting RNA to that obtained using a commercial phenol-based reagent. Our results demonstrate that this protocol is applicable to other plant species, including monocots, and offers yield and purity at least comparable to those provided by commercial phenol-based reagents.

**Conclusions:**

Here, we show that this previously published RNA isolation protocol can be easily extended to other plant species without further modification. Due to its simplicity and the use of inexpensive reagents, this protocol is accessible and affordable and can be easily implemented to work on different plant species in laboratories worldwide.

**Electronic supplementary material:**

The online version of this article (doi:10.1186/s13104-015-1119-7) contains supplementary material, which is available to authorized users.

## Findings

The isolation of good quality RNA in sufficient amounts is often a prerequisite for the analysis of gene expression, an approach that is widely used in laboratories worldwide routinely. However, most published protocols are labour intensive and/or use hazardous chemicals, and commercially available reagents or kits usually use toxic chemicals and are costly [1-3]. These constrictions can make RNA isolation a bottleneck for gene expression analysis in many laboratories, especially when involving large numbers of samples.

Oñate-Sánchez & Vicente-Carbajosa published an improved RNA isolation protocol for Arabidopsis that is simple, efficient, affordable, and avoids the use of toxic volatiles [4]. This article has been highly accessed, and the protocol successfully implemented in laboratories working on this model plant. However, a growing body of plant biologists work on other model or non-model species, including crops, and therefore information about the wide applicability of this or a similar protocol would be highly beneficial to a large number of researchers.

We set out to determine (i) whether the RNA isolation protocol described by Oñate-Sánchez and Vicente-Carbajosa [4] (see Additional file 1) can be applied to other plant species, and (ii) how the performance of this protocol compares to that of commercial phenol-based ready-to-use reagents. With this aim, we systematically extracted RNA from (a) Arabidopsis rosette leaves, (b) wheat leaves and (c) tomato leaves, using the protocol by Oñate-Sánchez and Vicente-Carbajosa [4], which uses non-toxic buffers containing sodium citrate and citric acid, and a commercial phenol-based reagent (TRI reagent, SIGMA, following the manufacturer’s instructions) in parallel. The resulting RNA samples were subjected to spectrophotometric quantification using nanodrop (NanoDrop 8000, Thermo Scientific), and the RNA absorbance ratios (A_260/A230_ and A_260/A280_) were obtained (Table 1, Additional file 2: Figure S1). Five hundred theoretical nanograms of RNA, according to the spectrophotometric quantification, were then loaded on a 1.5% agarose gel in TBE with in-gel ethidium bromide staining, alongside with an RNA ladder (0.5-10Kb RNA ladder, Life Technologies), in order to check the integrity of the isolated nucleic acids.

**Table 1:** **Spectrophotometric determination of total RNA quantity and quality**

	**TRI reagent**			**Oñate-Sánchez and Vicente-Carbajosa, 2008**		
**Samples**	**Concentration (ng/μL)**	**A260/280**	**A260/230**	**Concentration (ng/μL)**	**A260/280**	**A260/230**
Arabidopsis 1	355.2	2.12	1.91	613.3	2.11	2.36
Arabidopsis 2	497.4	2.00	1.90	501.6	2.03	2.19
Arabidopsis 3	231.7	2.05	1.65	481.3	2.10	2.18
Tomato 1	404.7	2.11	2.06	286.0	2.00	2.07
Tomato 2	382.9	1.99	1.88	458.3	2.12	2.09
Tomato 3	340.0	2.11	1.95	592.3	2.07	2.16
Wheat 1	238.0	2.07	1.51	379.1	2.08	2.20
Wheat 2	341.5	2.12	1.40	581.1	2.15	2.33
Wheat 3	332.5	2.10	2.02	340.0	2.07	2.24

RNA concentration and absorbance ratios (A_260/A230_ and A_260/A280_) are indicated per sample and extraction method. Samples were extracted and analysed in triplicates, as indicated. Starting material per sample was as follows: Arabidopsis leaves – eight leaf discs (7 mm diameter) of five-week-old plants grown in short day conditions; wheat leaves – eight leaf discs (7 mm diameter) of four-week-old plants grown in the glasshouse; tomato leaves – eight leaf discs (7 mm diameter) of five-week-old plants grown in the glasshouse. Samples were resuspended in 30 μL of water in all cases.

As shown in Table 1, in our hands, the theoretical concentration of RNA obtained from a given sample (Arabidopsis, tomato or wheat) was slightly higher when using the procotol by Oñate-Sánchez and Vicente-Carbajosa [4], according to the spectrophotometric quantification. Additionally, nucleic acid purity was higher in the RNA samples obtained following this protocol: while the A_260/A230_ and A_260/A280_ ratios were above 2.0 for these samples, representing high purity, in all cases, the A_260/A230_ value in the samples obtained using TRI reagent was generally lower (ranging between 1.40 and 2.06), indicating polysaccharide or, most likely, polyphenol contamination. Of note, it is known that traces of phenol contaminants can strongly inhibit downstream steps in genomic approaches, therefore compromising the results. Additionally, high absorbance at A_230_ can lead to the overestimation of RNA concentration in the sample.

**Figure 1:**
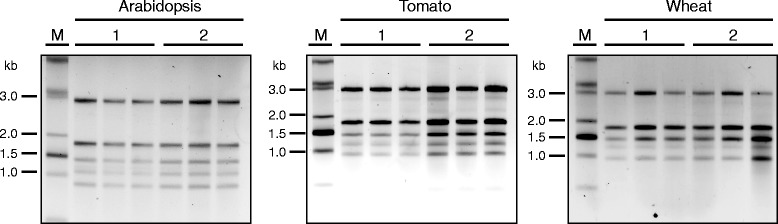
Non-denaturing agarose gel (1.5%) electrophoresis in TBE with in-gel ethidium bromide staining of RNA isolated from Arabidopsis, wheat and tomato leaves using TRI reagent (SIGMA) (labelled as 1) or the protocol by Oñate-Sánchez and Vicente-Carbajosa [4] (labelled as 2). In all cases, 500 theoretical nanograms, according to the spectrophotometric quantification, were loaded per lane. Samples were extracted and analysed in triplicates. Starting material per sample was as follows: Arabidopsis – eight leaf discs (7 mm diameter) of five-week-old plants grown in short day conditions; wheat – eight leaf discs (7 mm diameter) of four-week-old plants grown in the glasshouse; tomato – eight leaf discs (7 mm diameter) of five-week-old plants grown in the glasshouse. Samples were resuspended in 30 μL of water in all cases. The RNA ladder is the 0.5-10 Kb RNA ladder (Life Technologies).

Samples obtained from all three species using either protocol yielded RNA which was good quality (Figure 1). Of note, even though the same theoretical amount of RNA (0.5 μg) was loaded in all lanes, samples obtained using the commercial reagent usually appeared slightly fainter, indicating an overestimation of the RNA concentration most likely due to high absorbance at A_230_.

Our results demonstrate that the improved RNA isolation protocol published by Oñate-Sánchez and Vicente-Carbajosa [4] can be successfully applied to other plant species, including monocots, without further modification. Besides tomato and wheat, we have also successfully applied this method to *Citrus reticulata*, *Citrus limon*, *Solanum tuberosum*, *Solanum americanum*, *Amaranthus viridis* and *Malva parviflora* (Ana Arruabarrena, unpublished). Moreover, this protocol yields RNA whose quality is at least comparable to that provided by commercially available phenol-based reagents (Table 1, Figure 1), and the resulting nucleic acids can be directly used in downstream applications. Its simplicity and the low cost of materials used make this protocol widely accessible; therefore, it can be easily implemented in laboratories worldwide, allowing affordable and easy RNA isolation from different plant species.

## Additional files

Additional file 1: **Supplementary materials for plant RNA extraction protocol.**

Additional file 2: Figure S1. Absorbance curves obtained for the samples shown in Figure 1 and Table 1. **Method 1.** TRI Reagent method; **Method 2.** Protocol from Oñate-Sánchez & Vicente-Carbajosa (2008) [4].
