# Supplementary material for: Broad application of a simple and affordable protocol for isolating plant RNA
Source: BMC Res Notes. 2015 Apr 16;8:154. doi: 10.1186/s13104-015-1119-7 (PMC4404699; doi:10.1186/s13104-015-1119-7)
Supplement: Supplementary file 1 — Supplementary materials for plant RNA extraction protocol. [file 13104_2015_1119_MOESM1_ESM.docx]

**PLANT RNA EXTRACTION PROTOCOL**

(Adapted from Oñate-Sánchez and Vicente-Carbajosa, 2008, BMC Research Notes)

*Cautionary notes:*

- *All steps should be carried out at 4°C unless stated otherwise.*
- *Working solutions can be stored at RT for up to 1 month. Do not keep Cell Lysis Solution cold to prevent precipitation of SDS.*
- *Stock solutions can be stored at room temperature for more than 6 months.*

*Starting material (examples):*

- *Arabidopsis leaves: eight leaf discs (7 mm diameter) of five-week-old plants.*
- *Arabidopsis seedlings: four in vitro-grown two-week-old seedlings.*
- *Tomato leaves: eight leaf discs (7 mm diameter) of five-week-old plants.*
- *Wheat leaves: eight leaf discs (7 mm diameter) of four-week-old plants.*

1. Grind samples thoroughly.

2. Add 300 µl of Cell Lysis Solution and homogenize rapidly (vortex, tap and invert the tube); incubate at RT for 5 min.

3. Add 100 µl of Protein-DNA Precipitation Solution and homogenize (tap and invert the tube); incubate on ice for 10 min.

4. Centrifuge at 16,000 *g* for 15 min; transfer supernatant (300 µl) to a new tube.

5. Add 300 µl of isopropanol and homogenize (invert the tube); centrifuge at 16,000 *g* for 5 min and pour off supernatant.

6. Wash the pellet with 300 µl 70% ethanol (invert the tube); centrifuge at 16,000 *g* for 1 min and pour off supernatant.

7. Air-dry the pellet for 5 min or until there is no visible trace of ethanol.

8. Resuspend the pellet in 25 µl of RNase-free water.

Note: At this point, both DNA and RNA have been isolated. For downstream applications of the RNA, DNase treatment is required. A standard DNase treatment is shown below.

**DNase treatment (optional)**

a) Add 3 µl of 10x DNase buffer and 2 µl (2U) of DNase I (RQ1 RNase-free DNase, Promega M6101); incubate 30 min at 37°C.

b) Add 70 µl of RNase-free water, 50 µl of 7.5 M NH_4_Ac and 400 µl of 100 % ethanol; mix by inverting the tube. Centrifuge at 16,000g for 20 min.

c) Wash the pellet with 400 µl of 70% ethanol and centrifuge at 16,000g for 1 min. Pour off supernatant and air-dry for 5 min or until there is no visible trace of ethanol.

d) Resuspend in 30 µl of RNase-free water and store at -20°C.

e) Check for gDNA contamination by performing a PCR on your RNA samples. In some cases, the treatment described above might not be sufficient to degrade all the gDNA present in the sample. If gDNA is detected, repeat the procedure.

**SOLUTION RECIPES**

**Cell Lysis Solution (10 ml):**

2% SDS ………………………………………………………………….. 2 ml (10% stock pH= 7.2)

68 mM sodium citrate ……………………………………..….… 1.36 ml (0.5 M stock)

132 mM citric acid ……………………………………………..….. 1.32 ml (1 M stock)

1 mM EDTA …………………………………………………….….…. 20 µl (0.5 M stock)

(Note: The pH of the solution should be between 4 and 4.5; low pH is important to inactivate RNases.)

**Protein-DNA Precipitation Solution (10 ml):**

4 M NaCl ……………………………………………………..…………. 8 ml (5 M stock)

16 mM Sodium citrate ……………………………………..……. 320 µl (0.5 M stock)

32 mM Citric acid …………….……………………………………. 320 µl (1 M stock)

**Stock Solutions:**

1 M Citric acid (100 ml) ………………………..…………………. 19.22 g (MW= 192.124 g/mol)

0.5 M (tri)Sodium citrate dihydrate (100 ml) ………….. 14.706 g (MW=294.10 g/mol)

5 M NaCl (100 ml) …………………………..…………………..….. 29.22 g (MW= 54.44 g/mol)

7.5 M NH_4_Ac (100ml) …………………………………..….………. 58.37 g (MW= 77.083 g/mol)
