# Supplementary material for: Broad application of a simple and affordable protocol for isolating plant RNA
Source: BMC Res Notes. 2015 Apr 16;8:154. doi: 10.1186/s13104-015-1119-7 (PMC4404699; doi:10.1186/s13104-015-1119-7)
Supplement: Supplementary file 2 — Absorbance curves obtained for the samples shown in Figure 1 and Table 1. Method 1. TRI Reagent method; Method 2. Protocol from Oñate-Sánchez & Vicente-Carbajosa (2008) [4]. [file 13104_2015_1119_MOESM2_ESM.pdf]

Method 1

	Active	#	Well	Sample ID	Sample #	nm 1 abs.	A-260	A-280	260/280	260/230	ng/ul
Arabidopsis	■	1	C1	At1	7	8.861	8.881	4.187	2.12	1.91	355.2
	■	1	D1	At2	7	12.41	12.44	6.214	2.00	1.90	497.4
	■	1	E1	At3	7	5.780	5.792	2.826	2.05	1.65	231.7
Tomato	☐	1	C1	T1	1	10.091	10.117	4.787	2.11	2.06	404.7
	☐	1	D1	T2	2	9.553	9.573	4.810	1.99	1.88	382.9
	☐	1	E1	T3	1	8.479	8.500	4.035	2.11	1.95	340.0
Wheat	☐	1	F1	W1	1	5.936	5.950	2.873	2.07	1.51	238.0
	☐	1	G1	W2	1	8.519	8.537	4.022	2.12	1.40	341.5
	☐	1	H1	W3	1	8.294	8.311	3.957	2.10	2.02	332.5

Method 2

	Active	#	Well	Sample ID	Sample #	nm 1 abs.	A-260	A-280	260/280	260/230	ng/ul
Arabidopsis	☐	1	C1	At 1	5	15.285	15.331	7.252	2.11	2.36	613.3
	☐	1	D1	At 2	5	12.509	12.539	6.188	2.03	2.19	501.6
	☐	1	E1	At 3	5	12.005	12.031	5.718	2.10	2.18	481.3
Tomato	☐	1	C1	T1	7	7.140	7.151	3.567	2.00	2.07	286.0
	☐	1	D1	T2	6	11.429	11.457	5.405	2.12	2.09	458.3
	☐	1	E1	T3	6	14.747	14.807	7.149	2.07	2.16	592.3
Wheat	☐	1	F1	W1	5	9.457	9.478	4.565	2.08	2.20	379.1
	☐	1	G1	W2	5	14.496	14.529	6.748	2.15	2.33	581.1
	☐	1	H1	W3	4	8.481	8.500	4.107	2.07	2.24	340.0
